# Sleep Consequences of Prader-Willi Syndrome

**DOI:** 10.1007/s11910-023-01254-6

**Published:** 2023-02-15

**Authors:** Reem Itani, Emily S. Gillett, Iris A. Perez

**Affiliations:** 1 Department of Pediatric Pulmonology and Sleep Medicine, Children’s Hospital Los Angeles, Los Angeles, CA USA; 2 Keck School of Medicine of the University of Southern California, Los Angeles, CA USA

**Keywords:** Prader-Willi syndrome, Hypoventilation, Sleep-disordered breathing, Growth hormone, Excessive daytime sleepiness, Narcolepsy

## Abstract

**Purpose of Review:**

This paper reviews how sleep is impacted in patients with Prader-Willi syndrome (PWS), focusing on sleep-related breathing disturbances and excessive daytime sleepiness (EDS).

**Recent Findings:**

Hypothalamic dysfunction may underlie several aspects of the PWS phenotype. Central sleep apnea (CSA) can persist beyond infancy. Nocturnal hypoventilation is common and may occur without central or obstructive sleep apnea (OSA). Adenotonsillectomy, a mainstay of OSA treatment, may cause velopharyngeal insufficiency. Growth hormone (GH) is considered safe, but close surveillance for OSA remains important. Cardiac autonomic dysfunction occurs during slow wave sleep and may increase the risk of cardiovascular events. EDS and narcolepsy are also common. Modafinil and pitolisant are treatment options currently being studied.

**Summary:**

Sleep disorders are prevalent in individuals with PWS. Sleep-related breathing disorders present as CSA in infancy and later in life as OSA and hypoventilation. GH therapy has improved the clinical outcomes of patients with PWS, but close surveillance and treatment for OSA is recommended. EDS can persist even after sleep-related breathing disorders are treated, and some individuals may even develop narcolepsy. Early recognition and treatment of sleep-related disorders may prevent morbidity and result in improved survival of patients with PWS.

## Introduction

Prader-Willi syndrome (PWS) is a genetic disorder that affects about 1/10,000 to 1/30,000 individuals [1, 2••]. It is caused by a deficient expression of imprinted paternal genes on the long arm of chromosome 15. This problem can result from interstitial microdeletion of the paternally inherited 15q11.2-q13, maternal uniparental disomy 15, or, more rarely, due to imprinting defects [3]. Common to all cases of PWS is lack of expression of the small nucleolar ribonucleic acid-116 (*SNORD116*), which may account for the PWS phenotypes [4]. *SNORD116* is also considered a candidate gene for the sleep disturbances experienced by patients with PWS [5].

Individuals with PWS present with infantile hypotonia that results in feeding difficulties and failure to thrive. This is followed in early childhood by excessive, difficult to control eating that often leads to morbid obesity. Characteristic craniofacial features include narrow bifrontal diameter, almond-shaped palpebral fissures, narrow nasal bridge, and thin upper vermillion with down-turned corners of the mouth [1, 2••]. Other features include growth hormone deficiency with subsequent short stature, small hands and feet, hypogonadism, learning difficulties, and challenging behavior patterns [2••].

Recent evidence suggests that PWS pathophysiology is related to hypothalamic dysfunction [6••]. The hypothalamus is essential for respiratory control and sleep/wake regulation. In this paper, we review how PWS impacts sleep in pediatric and adult patients, focusing on the etiology, pathophysiology, management options, and outcomes of sleep-related breathing disturbances. In addition, we address sleep–wake problems, focusing on excessive daytime sleepiness (EDS) and narcolepsy.

## Ventilatory Control and the Spectrum of Sleep-Related Breathing Disorders in PWS

For patients with PWS, respiratory disturbances, particularly during sleep, begin in childhood [7]. They are a significant cause of morbidity and mortality. Sleep-related breathing disorders in these patients include central sleep apnea, obstructive sleep apnea, nocturnal hypoxemia, and sleep-related hypoventilation. Patients with PWS have abnormal ventilatory responses to hypoxia and hypercapnia that contribute to the development and severity of their sleep-related breathing disorders [8].

## Abnormalities of Ventilatory Control

### Hypoxic Ventilatory Response

The hypoxic ventilatory response (HPVR) is controlled by peripheral chemoreceptors located in the carotid bodies that modulate respiratory rate and tidal volume via the central respiratory control centers. The HPVR increases minute ventilation to maintain blood oxygen levels in the normal range under hypoxic conditions and following desaturations related to apneic pauses [8].

Patients with PWS have absent or blunted responses to breathing subatmospheric oxygen (14% FiO_2_) when compared to healthy controls [9]. Those who mount an HPVR do so by increasing tidal volume rather than respiratory rate, the opposite of the responses in healthy control subjects [9]. Additionally, patients with PWS paradoxically increase, rather than decrease, their respiratory rate in response to breathing a higher concentration of oxygen [10].

### Hypercapnic Ventilatory Response

The hypercapneic ventilatory response (HCVR) describes the reaction of the respiratory system to elevated levels of CO_2_. During extended periods of hypoventilation, elevated levels of CO_2_ are sensed by peripheral chemoreceptors in the carotid bodies while central chemoreceptors respond to the associated decrease in pH of CSF and body fluids.

When HCVR was tested in subjects with and without PWS, all individuals showed some increase in ventilation in response to hypercapnia, which is the normal response. However, obese subjects with PWS had a blunted HCVR compared to BMI-matched obese controls and non-obese subjects with PWS [9].

## Central Sleep Apnea

Central sleep apnea (CSA) is commonly seen in infants and children with PWS up to 2 years of age [11, 12]. The American Academy of Sleep Medicine (AASM) defines central apneas in children as follows: a ≥ 90% decrease in airflow signal from baseline for two breaths’ duration associated with a subsequent arousal or oxygen desaturation of ≥ 3%. In infants under 1 year old, central apneas may also be scored if they are associated with episodes of bradycardia [13]. Central apneas lasting ≥ 20 s are scored regardless of if they are followed by an arousal or desaturation event. Periodic breathing (defined as 3 central apneas of 3-s duration separated by breaths of no more than 20 s) also count towards central apnea scoring [13].

In children with PWS, central apneas occur predominantly during REM sleep and can cause severe intermittent oxyhemoglobin desaturations in some patients [12]. A genotype phenotype investigation showed that PWS patients with chromosomal deletions had a higher incidence of central apneas associated with significant oxygen desaturations (≥ 10%) than patients whose PWS phenotype was caused by another molecular mechanism [14].

CSA may be due to delays in maturation of the central ventilatory control centers or to an abnormal apneic threshold [9]. Congenital adrenal insufficiency is present in up to 60% of patients with PWS and may be related to the increased incidence of CSA [15]. In most cases, CSA improves with time [16]. However, central apneas can persist beyond infancy and may occur concurrently with obstructive apneas or hypoventilation [17•].

## Obstructive Sleep Apnea

Obstructive sleep apnea (OSA) is common in patients with PWS, particularly after 2 years of age [11], and is highly prevalent in adolescents and adults [18]. Sleep-related symptoms include consistent snoring, pauses in breathing for longer than 5 s, or daytime sleepiness, particularly during incurrent respiratory illness. Some patients are reported to sleep with their necks extended to relieve upper airway obstruction [19]. Patients with PWS have increased risk of OSA for many reasons, including obesity, hypotonia, micrognathia, small oronasopharynx, and viscous secretions [20]. Hypothyroidism, a known cause of OSA, is also seen in some patients with PWS [2••].

The prevalence of OSA in patients with PWS is approximately 80% and many patients are classified as having severe OSA [21, 22•]. Abnormal arousal threshold and arousal responses to hypercapnia and hypoxemia [23, 24] likely contribute to prolonged obstructive apneas and predispose patients to developing sleep related hypoventilation. In a study of 60 adults with PWS, 30% of patients had a sleep-related breathing disorder, most of which were OSA; of these, 8% of patients had previously undiagnosed hypoventilation and only half the cases of OSA were previously identified and treated [25]. Thus, many adult patients with PWS and OSA may not exhibit obvious symptoms, indicating the need for ongoing testing and surveillance in this high risk population.

## Sleep-Related Hypoventilation and Hypoxemia

Sleep-related hypoventilation is common in patients with PWS and can occur in absence of either CSA or OSA [17•]. This problem can be related to hypothalamic dysfunction and abnormal respiratory drive, obesity, hypotonia, scoliosis, or some combination of these factors. Hypoventilation out proportion to the degree of OSA has also been reported in children with PWS up to 14 years old [26•]. Hypoventilation is most prominent in REM sleep and may be associated with central hypopneas [17•]. Due to sleep-related hypoventilation, surveillance polysomnography with capnography is required in these patients.

Patients with PWS also have sleep-related hypoxemia, low oxygen desaturation nadirs, and high oxygen-desaturations indices. A genotype–phenotype correlation study showed those patients with PWS due to a chromosomal deletion had on average lower oxygen levels during sleep than patients with PWS due to other genetic mechanisms [22•].

## Evaluation of Sleep-Related Breathing Disorders

Overnight polysomnography (PSG) is indicated for all patients diagnosed with PWS to detect sleep-related breathing disorders. The PSG must be performed and interpreted per AASM scoring for age [13]. For all age groups, CO_2_ monitoring is essential due to the high prevalence of nocturnal hypoventilation. PSG is important prior to initiating growth hormone (GH) treatment, and periodically during GH therapy, as GH can exacerbate adenotonsillar hypertrophy [27, 28]. Patients may not report obvious symptoms of OSA [25, 29], making routine screening more important.

## Treatment of Sleep-Related Breathing Disorders

Once OSA is diagnosed, prompt referral to otolaryngology is recommended. Adenotonsillectomy (AT) is often the first-line treatment for OSA in children. A lateral neck X-ray may be helpful to assess adenotonsillar hypertrophy. OSA rarely resolves spontaneously in patients with PWS, so “watchful waiting” is not recommended. In a 4-year follow-up study of 20 children with PWS and OSA, only 2 children showed spontaneous resolution of their OSA [30]. After AT, children with PWS may have significant reduction in their obstructive apnea hypopnea index (oAHI); however, in many cases, the post-surgical oAHI remains elevated [31] and adjunctive therapy for OSA is required. It is therefore important to complete another PSG after AT to determine if a patient has persistent OSA [32••, 33].

Patients with PWS (especially young children and those with severe OSA, or morbid obesity) are at risk for acute post-operative complications and require close monitoring after AT [32••]. Velopharyngeal insufficiency is a rare complication of AT and may be prudent to counsel patients regarding this and consider speech evaluations before and after AT [32••].

Second-line therapy for OSA is most often continuous positive airway pressure (CPAP) or noninvasive positive pressure ventilation (NPPV) which improves residual OSA when used consistently during sleep [34, 35]. Significant behavioral problems are common in patients with PWS that can make adherence to PAP therapy quite challenging; therefore, we recommend incorporating behavioral therapy to assist with PAP desensitization and improve utilization.

Other components of the workup for OSA include TSH and free T4 levels to screen for hypothyroidism, and echocardiogram to look for pulmonary hypertension (particularly when severe OSA or sleep-related hypoventilation is present) [36]. Patients should also be screened for symptoms of gastroesophageal reflux disease (GERD) as it can worsen OSA [37]. GERD is common in patients with PWS, with a reported incidence of 53% [38].

While NPPV has a back-up rate and is an option for older patients with persistent CSA, NPPV is not usually practical for treating infants with central apneas. Instead, supplemental oxygen is often used as a therapy for central apneas in infants. In one study, oxygen administered to infants with PWS improved their Central Apnea Index (CAI) from median of 4.7 events/h to 2.5 events/h [12] and comparable results have been seen in subsequent studies [11].

**Fig. 1:**
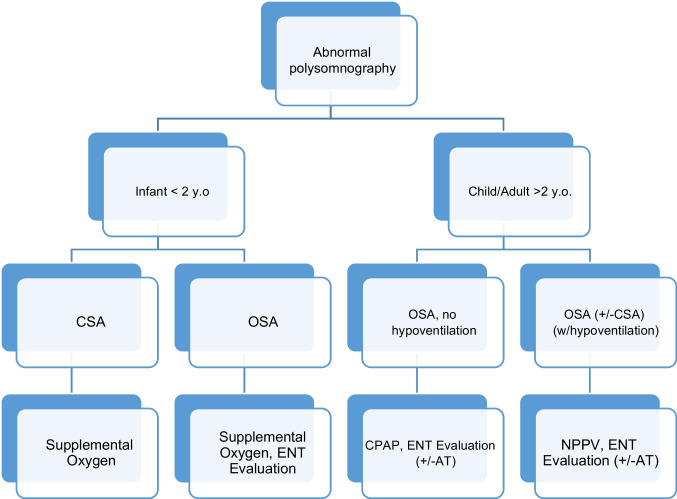
Respiratory management of sleep disordered breathing

An approach to respiratory management is offered in Fig. 1.

### Growth Hormone Therapy

GH is an FDA-approved treatment for individuals with PWS. It has been postulated to improve resting ventilation and ventilatory response to CO_2_ by modulating either the peripheral chemoreceptors sensitivity to CO_2_ or how input is relayed from peripheral chemoreceptors to central respiratory control centers [39].

It was previously suggested that GH therapy may exacerbate OSA due to accelerated growth of lymphoid tissues [27, 28]. More recent reports found no correlation between GH initiation and symptom onset [40] or any clinically significant negative impact on respiration [41•]. In a 2-year, randomized, double-blind, placebo-controlled cross-over study, it was found that there was no significant difference in AHI, CAI, or obstructive apnea index (OAI) in individuals who received GH compared with placebo, affirming the safety of GH therapy [42•]. A cohort of 19 adults who were overweight and with mild or no OSA at baseline who received GH treatment for 1 year showed improvement in sleep efficiency [41•]. In this group, longer apneas and increased oxygen desaturations were reported, but these findings were inconsistent. Most importantly, no deaths were reported [41•].

GH therapy can result in improvement in ventilatory control and inspiratory drive [39]. In an Australian multicenter retrospective study, there was no significant increase in median oAHI following initiation of GH in children with PWS [43]. One study showed worsening of sleep disturbance after initiation of GH therapy; however, on average, affected patients had higher IGF-1 levels and respiratory issues, so they may have had other causes of morbidity [28]. Nonetheless, because a high rate of sleep disordered breathing is present in patients with PWS, providers should screen patients for OSA with PSG prior to initiation of GH [44]. Given the benefits of GH in patients with PWS, every attempt should be made to treat OSA with the goal of continuing GH therapy. Current guidelines advise that clinicians should repeat a PSG 6 to 10 weeks after GH initiation, particularly in children (who may not demonstrate obvious symptoms of sleep apnea) and in patients whose breathing-related symptoms worsen [28, 45]. Other groups recommend repeating PSG within the first 3–6 months of GH initiation [44] as well as annually during continued GH treatment depending on evolving symptoms and clinical presentation [46]. One study found the development of moderate to severe OSA in 13% of children after the initiation of GH, supporting the need for surveillance PSG after starting treatment [43].

## Consequences of Sleep-Related Breathing Disorders

The estimated mortality rate of individuals with PWS is 1 to 4% per year and respiratory failure is the leading cause of death for all age groups [47]. Some deaths are reportedly related to sleep apnea or hypoventilation [19, 48]. There is also an increased risk of death from cardiopulmonary causes in those with the maternal disomy 15 subtype, although the mechanism for this is unknown [47]. Pulmonary hypertension, a known consequence of untreated OSA, is also reported in patients with PWS [49]. Other consequences of untreated OSA include increased risk of cardiovascular events and metabolic disease [50]. Adrenal insufficiency may be a contributing factor to sudden death, especially during periods of acute stress and infection [15]. Stress may also increase the frequency of central apneas in patients with PWS [51].

The behavioral problems exhibited by patients with PWS may be secondary to their sleep disorders, but causation has not been established. One study showed that increased OSA severity was associated with autistic-like behavior and impulsiveness in children with PWS [52]. While these features may be due to PWS itself, they may be worsened by OSA, which is known to increase daytime agitation and impulsiveness in children.

## Cardiac Autonomic Control During Sleep

Children with PWS have impaired parasympathetic modulation during slow wave sleep, resulting in high heart rates in absence of cardiovascular disease and independent of obesity [53••]. This is important as cardiac death is the second most common cause of mortality in patients with PWS [47]. Patients with PWS should have regular cardiovascular assessments, starting in childhood [53••, 54].

## Excessive Daytime Sleepiness and Narcolepsy

EDS unexplained by sleep disordered breathing is reported in some patients with PWS. Among respondents in the Global PWS Registry, 55% reported having EDS or narcolepsy. 6.1% of participants in the registry reported carrying a diagnosis of narcolepsy and 8.8% carried a diagnosis of cataplexy [55••]. While OSA is known to be associated with EDS, there is increasing evidence that it is not the only contributor in patients with PWS. Notably, some patients with PWS have persistent EDS even after weight loss and improvement in sleep disordered breathing, suggesting that primary hypersomnia is a characteristic feature of PWS [56]. Hypothalamic dysfunction may be a cause in some patients [57].

Some individuals with PWS have a narcolepsy-like presentation with sleep-onset REM periods, and sometimes cataplexy [58]. Low levels of orexin (also called hypocretin), a neurotransmitter produced in the hypothalamus that regulates appetite and sleep, is implicated in narcolepsy type I (formerly called narcolepsy with cataplexy). This disease is caused by the degeneration of hypothalamic neurons that produce orexin [59], resulting in very low or undetectable levels of orexin in the CSF. Interestingly, patients with PWS who exhibit EDS also have lower levels of orexin compared to normal individuals, but not as low as those with narcolepsy type I [60]. As opposed to the pathophysiology of narcolepsy type I, the number of hypothalamic orexin neurons is not reduced in individuals with PWS [61], suggesting that it is disruptions in the pathways connecting these hypothalamic neurons to the brainstem and cerebral cortex that are responsible for the lower orexin levels seen in these patients [62]. One study, showed that hypoxemia and hypercarbia inhibited hypothalamic orexin neurons in rats, suggesting that sleep disordered breathing may increase the risk of developing EDS [63]. Recognition and treatment of sleep disordered breathing is therefore of utmost importance to prevent further degradation of what may be an already impaired neuronal signaling system.

Diagnosing narcolepsy in patients with PWS can be challenging, especially as the diagnosis requires exclusion (or treatment) of other confounding sleep disorders, such as OSA. Patients with PWS sometimes have difficulty with adherence to PAP therapy and their OSA may never truly resolve, which leads to challenges making the diagnosis of narcolepsy based on current clinical guidelines. A Multiple Sleep Latency Test (MSLT) should be performed in all patients who have significant hypersomnolence out of proportion to their OSA. Ideally, the MSLT should be completed after patients have been established on appropriate PAP therapy, and PAP should be used during the overnight PSG preceding MSLT testing.

It is also essential to measure a wake CO_2_ level by arterial or capillary blood gas in any patient with PWS, obesity, and hypersomnia. Chronic respiratory failure with hypoventilation can cause EDS, headaches, and other symptoms, as well as contribute to cardiovascular stress and pulmonary hypertension. When daytime CO_2_ levels are significantly elevated (e.g., pCO_2_ > 50 mmHg), initiation of NPPV may be indicated. Blood gas testing can expedite the diagnosis of chronic hypoventilation compared to waiting for completion of PSG.

Modafinil, a central stimulant, may be helpful in the treatment of patients with PWS and EDS in the absence of sleep disordered breathing. An open-label pilot study of 10 patients with PWS and EDS showed significant improvement in sleepiness symptoms using the Epworth Sleepiness scale [64]. Pitolisant may also improve the EDS and neurospsychiatric symptoms in PWS patients by acting on the H3 receptor to potentiate the central activity of histaminergic neurons [65, 66•]. At the time of this review, the use of pitolisant for EDS in patients is not yet FDA-approved.

## Conclusions

Sleep disorders are prevalent throughout the lifetime of individuals with PWS. Sleep-related breathing disorders present as CSA in infancy and OSA and hypoventilation in older children, adolescents, and adults. GH therapy has improved the clinical outcomes of patients with PWS, but close surveillance for the development or worsening of OSA is of utmost importance. EDS can persist even after sleep-related breathing disorders are treated and some individuals may develop narcolepsy. Early recognition and treatment of sleep disorders may prevent and improve survival of patients with PWS and is therefore a critical component for medical management for these patients.
